# Use of globally unique identifiers (GUIDs) to link herbarium specimen records to physical specimens

**DOI:** 10.1002/aps3.1027

**Published:** 2018-03-07

**Authors:** Gil Nelson, Patrick Sweeney, Edward Gilbert

**Affiliations:** ^1^ iDigBio Florida State University 142 Collegiate Loop, P.O. Box 3062664 Tallahassee Florida 32306‐2664 USA; ^2^ Division of Botany Peabody Museum of Natural History Yale University P.O. Box 208118 New Haven Connecticut 06520 USA; ^3^ School of Life Sciences Arizona State University P.O. Box 874501 Tempe Arizona 85287 USA

**Keywords:** digitization, globally unique identifer (GUID), herbarium, herbarium specimen, identifier, recommended practices

## Abstract

With the advent of the U.S. National Science Foundation's Advancing Digitization of Biodiversity Collections program and related worldwide digitization initiatives, the rate of herbarium specimen digitization in the United States has expanded exponentially. As the number of electronic herbarium records proliferates, the importance of linking these records to the physical specimens they represent as well as to related records from other sources will intensify. Although a rich and diverse literature has developed over the past decade that addresses the use of specimen identifiers for facilitating linking across the internet, few implementable guidelines or recommended practices for herbaria have been advanced. Here we review this literature with the express purpose of distilling a specific set of recommendations especially tailored to herbarium specimen digitization, curation, and management. We argue that associating globally unique identifiers (GUIDs) with physical herbarium specimens and including these identifiers in all electronic records about those specimens is essential to effective digital data curation. We also address practical applications for ensuring these associations.

The advent of the U.S. National Science Foundation's (NSF) Advancing Digitization of Biodiversity Collections (ADBC) program has fostered an accelerated rate of herbarium specimen digitization in the United States, through both internally and externally funded initiatives. Collectively, U.S. herbaria curate approximately 76 million specimens, or about 20% of the world's herbarium holdings (Thiers, 2017). Although the iDigBio database, which includes U.S. and non‐U.S. herbarium specimen records, currently includes a modest 48 million (12.6%) of these worldwide specimen records and about 17% of U.S. records, these numbers are destined to grow substantially over the next few years as digitization initiatives continue to expand, NSF‐funded Thematic Collections Networks (TCNs) ramp up data mobilization, and the NSF continues to require award recipients to contribute data to the iDigBio data store. Of the approximately 641 active U.S. vascular and non‐vascular plant herbaria reported by Index Herbariorum (Thiers, 2017), about 266 (41%) (iDigBio, 2017) are currently participating in at least one of the nine ADBC‐funded, botany‐focused TCNs, almost all of which are actively digitizing their collections (https://www.idigbio.org/content/thematic-collections-networks). Recent evidence from 116 self‐defined small herbaria suggests that 84% of these are databasing their collections and 48% are imaging them (iDigBio, unpublished survey data, 2015).

As the numbers of electronic records for herbarium specimens and the aggregation of these records proliferate on the internet, the importance of the unambiguous identification of these records, as well as linking these records to the physical specimens they represent and related data from other sources, will intensify. Ideally (e.g., see Hugo et al., 2017; McMurry et al., 2017), future internet searches (by a human or software agent) will return not only one or more records of a specimen's label data and physical location, but also links to data about separately housed genetic resources, taxonomic concepts, related literature and source materials (e.g., catalogs, ledgers, field notes), duplicates deposited in other herbaria, additional preparations (e.g., fruits stored separately, multiple sheets), enriched data appended post‐collection (e.g., georeferences, determinations, morphological measurements, phenological scorings, species associations, and other annotations), and potentially other objects or metadata derived from and directly related to the specimen, all of which will be interconnected through the Semantic Web (Berners‐Lee et al., 2001; Berners‐Lee, 2009). Here we argue that facilitating this linking through the association of globally unique identifiers (GUIDs) with physical herbarium specimens and including these identifiers in all electronic records about those specimens is essential to effective digital curation. As noted below, this does not necessarily imply that a GUID label must be affixed to each physical specimen, only that an identical and persistent GUID be included in any and all database records created for that specimen and its derivatives (e.g., genomic records, images, fruits stored separately). Ensuring the association of a persistent GUID with every specimen will facilitate efficient discovery and foster linkages between the rapidly growing store of digitized data for research and other uses. A specimen‐level GUID should not be confused with a specimen record identifier. The specimen‐level GUID references the specimen itself, while a record identifier references a database record about the specimen.

In the interest of common definitions and best practices, here we address the issue of creating and assigning specimen‐level GUIDs to herbarium specimens. By specimen, we mean a specimen in the sense of the *International Code of Nomenclature for Algae, Fungi, and Plants* (ICN; McNeill et al., 2012), and we elaborate on this in the Discussion. With regard to publishing specimen‐level GUIDs, under Some Current Identifier Practices, below, we review and discuss current practices and the definitions and relationship between DWC:occurrenceID and DwC:materialSampleID for use as specimen‐level identifiers. We are not addressing the numerous additional GUID values that might appear in a single specimen record. Based on Darwin Core (DwC; Wieczorek et al., 2012; Biodiversity Information Standards [TDWG], 2018) terminology, records might also include GUID values for locationID, eventID, collectorID, taxonID, and others. These additional GUID values allow a specimen record to be linked to information about where a specimen was collected, when it was collected, and who collected it, as well as information about the taxon the specimen is presumed to represent.

This paper will provide: (1) a background for the application of GUIDs to herbarium specimens, (2) a review of relevant literature and workshop outcomes, (3) a summary of the desired properties of specimen‐level GUIDs, (4) practical guidelines for the application of specimen‐level GUIDs to herbarium specimens, and (5) inspiration to curators and collections managers to become engaged in the process of assigning and managing specimen‐level GUIDs.

## METHODS

We reviewed current philosophical, conceptual, and technical literature associated with the concept of GUIDs, extrapolated the conclusions most relevant to linking herbarium specimens across the internet, and narrowed these conclusions to practical guidelines for managing plant specimens in a herbarium. Additionally, we were informed and received feedback through workshops that were concerned with specimen digitization and related activities (e.g., Nelson et al., 2015).

Conceptually, the use of GUIDs to uniquely identify and link specimen objects with related electronic records is not new. A rich literature has developed within the biodiversity informatics and natural history collections communities over the past decade to address this topic (e.g., Page, 2008, 2009, 2016; Richards et al., 2011; Hyam et al., 2012; Hagedorn, 2013; Hagedorn et al., 2013; Miller et al., 2015; Guralnick et al., 2015). Although much of this literature focuses on pre‐implementation theory and recommendations, the conversion of theoretical constructs into implementable strategies is moving forward (e.g., Baskauf, 2010; Hyam et al., 2012) and the growing store of electronic specimen data makes practical application within the discipline urgent. Here we build on and extend these previous works to provide specific guidance to herbarium curators and collections managers for enabling the use of specimen‐level GUIDs. Although we briefly review the important body of literature that speaks to the necessity of GUIDs for biological specimens, our primary purpose is the distillation of this literature into a set of practical guidelines usable by herbarium curators and collections managers for implementing the use of specimen‐level GUIDs at an individual herbarium.

## RESULTS

Several important characteristics of GUIDs are clear from our review of related literature.

### Representation (What does the GUID represent?)

It is agreed that in order to connect various biocollections objects in the “cloud,” identifiers are needed for a variety of purposes (Richards, 2010). Features of biocollections objects that benefit from having GUIDs assigned include scientific names, taxonomic concepts, images, and specimens (Richards, 2010). In the case of specimen‐level identifiers, there is ambiguity and some disagreement both in the literature and in practice about what the identifiers should or do represent (e.g., a physical object, a digital object, an abstract concept/object) (Richards et al., 2011; iDigBio, 2013; Guralnick et al., 2015). Additionally, there is ambiguity in defining what exactly constitutes a specimen (Richards et al., 2011; Guralnick et al., 2015). This is especially true for aggregated objects, such as numerous fishes in a jar, several fossils in a single matrix, or fruits stored separately from a herbarium sheet. Regardless of what is represented, it is important that the reference is unambiguous (Richards et al., 2011; Guralnick et al., 2015).

### Persistence

Persistence is essential to the use of identifiers (Richards et al., 2011; iDigBio, 2013; Guralnick et al., 2015). Once assigned to an object or concept, a GUID should not be deleted or altered. Including a unique, persistent, specimen‐level GUID in all published records of a specimen makes it possible for search engines or other software agents to find and serve all published information about that specimen, including its physical location, owner, and derivatives. It is also important that the identifier remains unchanged in the canonical specimen database or collection management system. Some (Richards et al., 2011; McMurry et al., 2017) take the view that “persistent identifiers” imply resolvability.

### Uniqueness

Equally important as persistence, specimen‐level identifiers should be globally unique (Richards et al., 2011; Guralnick et al., 2015; McMurry et al., 2017). A particular specimen‐level GUID should be assigned to one and only one specimen, regardless of the lifespan or physical location of the specimen. Uniqueness does not imply that a specimen is restricted to a single identifier, although it is better to use existing identifiers rather than create new ones, when appropriate (Richards et al., 2011).

### Resolvability

A highly desirable characteristic of specimen‐level and other kinds of GUIDs is that they are resolvable (Page, 2009, 2016; Richards et al., 2011; Guralnick et al., 2014, 2015; McMurry et al., 2017), that is, the identifier can be used, via an internet service, to find out more about the asset that is identified (Richards et al., 2011; Page, 2016). Resolvable GUIDs are central to making data about the asset easily available on the internet and facilitate making linkages between assets in the cloud (Page, 2009, 2016; Richards et al., 2011). An example of a resolvable identifier with built‐in resolution (i.e., actionable or dereferenceable) is an HTTP uniform resource identifier (URI), which is the kind of identifier used in the Semantic Web. GUIDs without built‐in resolution can be resolved through stand‐alone resolution services. Examples of resolution services that make GUIDs resolvable include the International DOI Foundation's digital object identifier (DOI; http://www.doi.org/) and the California Digital Library's EZID (http://ezid.cdlib.org/). The DOI and EZID systems are especially well‐developed for storing, maintaining, and serving metadata about documents.

### Opacity

GUID values can be transparent or opaque (Page, 2009). Transparent values include those that contain human‐decipherable text or human‐meaningful strings. DwC triplets (e.g., Uconn:CONN:CONN00050395) and HTTP URI identifiers (e.g., http://herbarium.bio.fsu.edu/000002561) are examples of transparent identifiers that connote some sense of meaning, such as ownership, to the human user. Opaque identifiers (e.g., a universally unique identifer [UUID], e3ad9bb3‐cb8e‐475c‐aff5‐87f877b56120) contain no apparent human‐decipherable information and are construed strictly as meaningless strings. The lack of apparent meaning underscores their universality and reduces the likelihood that they will be altered or replaced (McMurry et al., 2017).

### Physical placement

We do not know of institutions that are attaching labels with opaque, UUID‐based GUIDs to specimens, although we do know of institutions using DwC triplets (i.e., institutionCode + collectionCode + catalogNumber) as the value of barcode labels attached to specimens (e.g., WIS‐L‐0037826). Among herbaria, it is widespread practice to attach catalog numbers, accession numbers, or other institutional identifiers to specimens. Often these values are represented in barcode format. These values typically provide locally unique identifiers that allow specimens to be referenced within a single herbarium or institution; however, they are commonly also referenced outside of the parent institution (e.g., in publications).

### Creating/minting GUIDs

In the literature, several methods have been discussed regarding where and by whom a GUID is created (Page, 2009). In most cases, the institution owning the specimen creates the specimen‐level GUID (E. Gilbert, personal observation). However, some have argued that specimen‐level GUIDs should be created in the field as specimens are collected (Guralnick et al., 2015). In terms of how GUIDs are minted, some databases have the ability to create GUIDs. For example, Specify (http://specifyx.specifysoftware.org) mints GUIDs as the ObjectID and Emu (https://emu.kesoftware.com) as a version 4 UUID in a designated GUID table at the time a new specimen record is created. Symbiota (Gries et al., 2014) can be configured to automatically generate version 4 UUIDs or allow the data owner to define an alternative specimen‐level GUID format (e.g., HTTP URI, Life Sciences Identifier [LSID]). For custom‐built solutions or databases, a number of programming and scripting languages can mint UUIDs (e.g., Java, Perl, PHP, Python, SQL, VBA). RFC 4122 (https://tools.ietf.org/html/rfc4122) and ISO/IEC 9834‐8:2014 (https://www.iso.org/standard/62795.html) specify standards for generating UUIDs. Within the ADBC TCNs (iDigBio, 2013), GUIDs are being generated in a variety of ways.

### Some current identifier practices

In the context of herbarium collections, plant material collected on the same date, in the same locality, and by the same primary collector are considered to share a common collecting event. Occasionally, a simple field number will be assigned as an identifier representing the event. It is common for the primary collector to assign a unique personal collection number (for example, in the format DwC recordNumber) to each taxon collected within an event. Specimens of the same taxon (i.e., all sharing the same collector number/recordNumber) are regularly distributed to multiple institutions as specimen duplicates and these institutions may give various kinds of identifiers to them (e.g., catalog numbers, accession numbers). It is very uncommon for any of these identifiers to be GUIDs and thus possess the desirable characteristics presented above. Specimen‐level GUIDs are typically assigned at a later stage, often in conjunction with databasing.

It is common practice that when herbarium specimen data are shared from the canonical database they are shared as Occurrences with a unique value in the DwC occurrenceID field, the latter serving as a proxy for a unique specimen identifier. Although this practice reflects current widespread community convention, it is at odds with a strict interpretation of Darwin Core, which defines occurrenceID as an identifier for the Occurrence, with an Occurrence being “an existence of an Organism (sensu http://rs.tdwg.org/dwc/terms/Organism) at a particular place at a particular time.” Depending on the properties being invoked, a herbarium specimen is at once an Occurrence and a materialSample, as each of these has overlapping properties. In many cases, an Occurrence could be considered equivalent to a herbarium specimen from a DwC perspective; however, in the case of duplicates from the same organism, technically all of the specimens represent the same Occurrence and thus should all have the same occurrenceID. Although as discussed below, it is often not feasible to assign the same occurrenceID to legacy specimens distributed across multiple institutions. Darwin Core includes a more recently posited term that could be equated with a physical specimen, materialSample, defined as “A physical results of a sampling (or subsampling) event,” and further explains that “In biological collections, the material sample is typically collected, and either preserved or destructively processed” (Biodiversity Information Standards [TDWG], 2018; http://rs.tdwg.org/dwc/terms/index.htm#MaterialSample). The term Occurrence was issued 19 November 2008, several years prior to the establishment of the term materialSample (issued 28 March 2013). Until this latter date, there was no field perfectly suited for a specimen‐level GUID, hence the community selected occurrenceID for this purpose. We do not disagree with the position that materialSampleID may be a more appropriate field for a specimen‐level identifier, although to our knowledge no herbaria are using this field to house a specimen‐level GUID, and an enormous data sharing and aggregation environment (e.g., Specify, Symbiota, GBIF, and iDigBio) has been built around using occurrenceID as a specimen‐level identifier.

## DISCUSSION

Although there is a rich literature regarding specimen‐level GUIDs and identifiers, there are few practical guidelines or best practices for incorporating specimen‐level GUIDs into herbarium curation and digitization workflows. Widespread adoption of common guidelines is essential to ensuring the consistent and proper use of GUIDs within the herbarium community.

There has been widespread agreement that assigning GUIDs to specimens is a critical step for mobilizing specimen data at a global scale (e.g., Page, 2008, 2009, 2016; Richards et al., 2011; Hyam et al., 2012; Hagedorn, 2013; Hagedorn et al., 2013; Miller et al., 2015; Guralnick et al., 2015). Details such as what specific collection entity a GUID represents and at which point of the workflow the GUID should be assigned have been highly debated. Convincing arguments have been presented for identifiers being assigned to an individual specimen, groups of specimen duplicates across institutions, and even individual organisms.

It is our view that each physical specimen within a collection should be associated with a GUID. We adopt an ICN concept of specimen.^1^ An implication of this stance is that each duplicate (in the ICN sense^2^ ) should have its own GUID. As discussed above and in keeping with widespread practice, we also think that when data are shared from the canonical database, each specimen should be treated as an Occurrence with a GUID included in the DwC occurrenceID field.

A strict reading of DwC definitions for occurrenceID, Occurrence, and Organism suggests that all members of a set of duplicates collected from the *same Organism* should have the same occurrenceID. However, this is problematic for two main reasons. First, many institutions that have been publishing specimen Occurrence data sets with occurrenceID values have been populating this field with an identifier that is assigned to the specimen or the authoritative specimen record in the canonical institutional database. A large data sharing and aggregation edifice (e.g., iDigBio and GBIF) has been built around this practice. Second, at this juncture, it is difficult in the case of duplicates derived from the same organism to ensure that all duplicates representing a DwC Occurrence are given the same occurrenceID. With legacy specimens, it is often difficult to discern whether duplicates represent material collected from a single individual (e.g., stem fragments from a single tree), multiple individuals (e.g., cuttings from several trees), or multiple individual plants. In fact, each separate specimen could contain multiple individuals mounted on a single sheet (e.g., small annual plants). Furthermore, it may take years for all the material of a single collecting event to be fully processed within all receiving institutions, and the final result is typically a set of related specimens of multiple taxa and individuals distributed among several institutions. Given that there is no widely adopted, agreed upon, easy‐to‐use method to discover existing identifiers, if they exist, within the herbarium community specimen‐level GUID assignments have commonly been limited to represent individual specimens within a single collection. Thus, we argue that the ICN concept of specimen conveniently aligns with databasing practices and the use of a distinct occurrenceID for each specimen within a collection. Our concept of specimen‐level GUID should not be confused with the other kinds of GUIDs that could be associated with a specimen, especially DwC organismID and collectingEventID GUIDs. We recognize that within some collection disciplines where the specimen entity is closely aligned with individual organisms (e.g., insects or mammals) this may be a subtle distinction. However, when it is definitely known that multiple herbarium specimens were collected from the same individual (e.g., shrub or tree) the specimens would share the same organismID, which allows for linking all specimens taken from a particular organism.

Specimen‐level GUIDs should be assigned to specimens by the herbarium that owns the specimens or holds and curates the specimens for another institution. The point at which to initially assign GUIDs to specimens is at or after the time they are mounted by the herbarium that will curate the specimens and before records about the specimens are published (but see Guralnick et al., 2015). Institutions and individuals that possess material on loan from another institution should avoid the temptation to assign specimen‐level GUIDs to these specimens. Instead, they should request GUID assignments directly from the loaning institution to ensure those used within any potential research match those in the source collection and database. Likewise, gifts and exchanges distributed pre‐curation (typically in paper, unmounted, and without a catalog number) should not be assigned a specimen‐level GUID by the distributing institution.

We are not arguing here that a label containing the specimen‐level GUID value, either embedded in a machine‐readable format or as human‐readable text, necessarily be attached to the physical specimen, although this is an option for specimens being newly accessioned or digitized. It has been widespread practice within the herbarium community to attach catalog numbers, accession numbers, or other institutional identifiers to specimens. Storing these values in the authoritative specimen record that also contains the specimen‐level GUID provides a way to associate the specimen‐level GUID with a single, physical specimen, even if a GUID label is not physically attached to the specimen. Nor are we arguing that existing catalog numbers or other institutional identifiers be replaced with GUIDs. Some herbaria have chosen to abandon previous series of catalog numbers, replacing them with newly minted barcode value series, and a few (e.g., the Florida Museum of Natural History [FLAS], the Australian National Herbarium [CANB]) are forcing newly minted barcode values to exactly match existing catalog numbers to ensure consistency, preserve existing series, and ensure fidelity with citations in previous publications.

Specimens may have multiple identifiers representing various purposes, but no specimen‐level GUID should be applied to more than a single specimen (sensu ICN) and the derivative preparations of that specimen (e.g., a leafy branch and fruit from a single Occurrence may be stored separately, but are still of a single Occurrence and will bear the same specimen‐level GUID). While assigning more than one specimen‐level GUID to a specimen record is not ideal (Guralnick et al., 2015) and we do not recommend it, this is a potential reality that must be supported by management software, publishing tools, and herbarium staff.

Several formats are available for populating GUID fields for biodiversity specimens (Table 1). Although many of these are being used by various herbaria and other biodiversity collections, here we focus largely on the use of UUID values. We note that some institutions have adopted the DwC triplet, as defined in DwC terms (Biodiversity Information Standards [TDWG], 2018; http://rs.tdwg.org/dwc/terms/index.htm#occurrenceID) for assigning GUIDs to specimens. A DwC triplet is a concatenation of the institution code, collection code, and catalog number in the form urn:catalog:[institutionCode]:[collectionCode]:[catalogNumber]. To facilitate global uniqueness, the Biodiversity Information Standards (TDWG) recommends prepending the namespace designation urn:catalog:. Hence, the identifier for a specimen at the Florida State University herbarium (FSU) with catalog number 123456789 would be urn:catalog:FSU:FSU:123456789. There has been controversy about the use of the DwC triplet due to its complexity, persistence, inconsistency, and potential for inadvertent duplication (Page, 2009; Guralnick et al., 2014). We further note that some institutions are using HTTP URI (Hyam et al., 2012) and LSID (Page, 2008; Pereira et al., 2009) identifiers, among others. However, HTTP URIs are not opaque and, according to Greg Whitbread (personal observation, 2017), the “TDWG Executive voted to an interim rewrite of the GUID applicability statement to take down the TDWG preferred status of LSID pending a full revision of the standard from the Persistent Identifiers Task Group.” In some instances, especially within a single institution, identifiers that are meaningful to humans are helpful. In other instances, such as when specimens permanently migrate from one herbarium to another (e.g., when an orphan collection finds a new home), meaningful identifiers can be problematic. The receiving herbarium might be tempted to alter, add, or replace the current identifiers, preferring instead a “local” identifier. Additionally, HTTP URIs imply resolvability, the latter of which may confuse some users when the links are unresolvable by design or when broken. Nevertheless, we do not recommend that such institutions eliminate or replace existing identifiers or necessarily convert to using UUID values, especially where currently used values satisfy the basic GUID requirements of uniqueness and persistence. For institutions just beginning to examine the use of GUIDs, we recommend strong consideration of UUID values as an institutional standard.

**Table 1:** Globally unique identifier formats

Identifier	Acronym	Resource	Example
Archival Resource Key	ARK	https://en.wikipedia.org/wiki/Archival_Resource_Key	
Darwin Core (DwC) triplet	DwC triplet	http://iphylo.blogspot.com/2011/12/dna-barcoding-darwin-core-triplet-and.html http://rs.tdwg.org/dwc/terms/#occurrenceID	UConn:CONN:CONN00050395
Digital object identifier	DOI	http://www.doi.org/	10.5063/AA/NRS.480.1
Hypertext Transfer Protocol Uniform Resource Identifier	HTTP URI	http://www.w3.org/Addressing/URL/uri-spec.html	
International GeoSample Number	IGSN	http://www.geosamples.org	IEPRI0285
Life Sciences Identifier	LSID	http://wiki.tdwg.org/twiki/bin/view/GUID/LSID	
Universally unique identifier	UUID	https://en.wikipedia.org/wiki/Universally_unique_identifier	e3ad9bb3‐cb8e‐475c‐aff5‐87f877b56120

Ideally, GUIDs should resolve to metadata about the object referenced (Page, 2009; Richards et al., 2011; McMurry et al., 2017). However, numerous challenges prevent this from being a requirement across all collections. To date, there is no comprehensive community‐adopted service that makes identifiers actionable for biodiversity specimens (Guralnick et al., 2014; Page, 2016). Many small‐ to medium‐sized herbaria lack the information technology infrastructure to establish a local resolution service for their collection. Even when services are established, the inconsistent financial and technical support that plague many biological collections can interfere with long‐term maintenance of a resolution service and associated domain names. It is best for institutions not to assume that resolvable GUID formats are a requirement, unless the maintenance of a long‐term, reliable resolution service is certain. However, it is inevitable that collections will assign purportedly resolvable GUIDs dependent on services that eventually are not maintained. Where resolution services are established, we recommend that a UUID value be used within any specimen‐level GUIDs that are assigned. The California Digital Library's Archival Resource Key (ARK) ID (ARK:99999:e3ad9bb3‐cb8e‐475c‐aff5‐87f877b56120) is an example of the latter format. Our recommendation is that only the UUID portion of such identifiers be assigned to occurrenceID and that the prefixed version be stored in DWC:references.

Many in the biodiversity informatics community strongly advocate for GUIDs that have built‐in resolvability. We anticipate that such a resolution service will be developed. However, we argue that postponing the assignment of GUIDs to specimens until such resolution service is available may be short sighted. In the interim, development of such a service necessitates the assignment of persistent GUIDs to all specimens and the inclusion of these identifiers in published specimen records is likely to encourage resolver development by the informatics community.

It is clear from the above that the assignment of GUIDs to physical specimens will impact standard curatorial and herbarium management practice and make it necessary for collections personnel to ensure the availability of specimen‐level GUIDs to all users of the specimens, whether in‐house or virtually via remote connectivity or distribution. For electronic or virtual use, this means ensuring that all data sets, whether distributed or downloaded, include the specimen‐level GUID value for every specimen (as the occurrenceID value), regardless of whether the specimen‐level GUID is requested by the user. For in‐house users and in the absence of specimen‐level GUID values physically attached to specimens, a connection to the canonical database that contains the specimen‐level GUID values should be accessible to onsite researchers and workers, and the use of these specimen‐level GUID values should be encouraged.

## CONCLUSIONS

We strongly recommend that GUID values should be associated with all specimens and included in digital records of those specimens. Our stance in this paper recommends creating a DwC Occurrence record for each specimen. With most herbarium specimens, this approach would not be a misapplication of the DwC standard—the exception would be when specimen duplicates are derived from a single organism. As discussed above, we do not see how this exception can be avoided, given current community dissemination and aggregation practices and given the difficulty of uniting widely dispersed legacy duplicates under a single Occurrence. Equally important, we argue that curators and collections managers should be aware of the great importance of specimen‐level GUIDs and be deeply engaged in promotion and implementation of workflows that incorporate the assignment of occurrenceID GUIDs. This is especially true given the importance of occurrenceID GUIDs to future research and discoverability as herbarium data sets are aggregated and commingled with data sets within which duplicate specimen records might occur.

Collections managers and other technical personnel are the individuals tasked with assigning and managing specimen‐level GUIDs and ensuring that these GUIDs are persistent and passed on to aggregators and end users as part of electronic data sets (as the occurrenceID). The process of assigning, managing, and sharing herbarium specimen‐level GUIDs requires several steps: (1) creating (or ensuring the existence of) a field for a globally unique specimen‐level identifier within the owning institution's canonical specimen database, (2) ensuring that the database field is constrained as unique, (3) minting identifiers in one of several formats, (4) associating an identifier with each specimen by populating the identifier column in the electronic record for that specimen, (5) maintaining identifier persistence by ensuring that the field cannot be edited, and (6) publishing identifiers along with specimen‐specific data to journals and aggregated databases.

Sophisticated databases can be configured to accomplish more than one of these steps concurrently and automatically.

### Recommended practices for minting, managing, and sharing GUIDs for herbarium specimens

1. We recommend assigning persistent, opaque specimen‐level GUIDs, with a preference for the UUID format.
2. We recommend strong consideration for creating a materialSampleID GUID for each specimen, with a preference for the UUID format.
3. GUIDs need not be affixed to the physical specimen, but an institutional database mechanism must exist to connect the physical specimen with the GUID.
4. Specimen records should be published as Occurrences with the DwC occurrenceID field populated with a specimen‐level GUID.
5. Specimen Occurrence records (and associated occurrenceID GUIDs) should be created by the herbarium that owns the specimens or holds and curates the specimens for another institution.
6. Specimen Occurrence records (and occurrenceID GUIDs) should be created at or after the time they are mounted by the herbarium that will curate the specimens and before records about the specimens are shared or published.
7. The original occurrenceID GUIDs should remain associated with the specimen, regardless of a change in institutional ownership of the specimen, or changes in collection management database platforms.
8. Herbaria currently using occurrenceID GUIDs in a format other than UUID should not replace existing GUID values with UUID values, but might consider UUID as a format for future GUID assignments.
9. If multiple occurrenceID GUIDs are assigned to records, the old and new values should be maintained and published when specimen records are shared.
10. A herbarium should inform its users that occurrenceID GUIDs should be cited in publications and data sets (e.g., genomic records, ecological data sets) along with other data traditionally used to cite specimens (e.g., institution, Index Herbariorum code, catalog number, collector, and collection number).
11. We recommend strong consideration for creating a materialSampleID GUID for each specimen, with a preference for the UUID format.

## Notes

1 From ICN Article 8.2, “For the purpose of typification a specimen is a gathering, or part of a gathering, of a single species or infraspecific taxon made at one time, disregarding admixtures (see Art. 9.14). It may consist of a single organism, parts of one or several organisms, or of multiple small organisms. A specimen is usually mounted on a single herbarium sheet or in an equivalent preparation, such as a box, packet, jar, or microscope slide.

2 From the ICN Article 8.3, “A specimen may be mounted as more than one preparation, as long as the parts are clearly labelled as being part of that same specimen. Multiple preparations from a single gathering that are not clearly labelled as being part of a single specimen are duplicates, irrespective of whether the source was one organism or more than one (but see Art. 8.5).”
